# Role of Exposure to Lactic Acid Bacteria from Foods of Animal Origin in Human Health

**DOI:** 10.3390/foods10092092

**Published:** 2021-09-04

**Authors:** Carla Miranda, Diogo Contente, Gilberto Igrejas, Sandra P. A. Câmara, Maria de Lurdes Enes Dapkevicius, Patrícia Poeta

**Affiliations:** 1 Microbiology and Antibiotic Resistance Team (MicroART), University of Trás-os-Montes and Alto Douro, 5000-801 Vila Real, Portugal; carlisabelmi@utad.pt (C.M.); ppoeta@utad.pt (P.P.); 2 Department of Veterinary Sciences, University of Trás-os-Montes and Alto Douro, 5000-801 Vila Real, Portugal; diogo.contente95@gmail.com; 3 Associated Laboratory for Green Chemistry (LAQV-REQUIMTE), University NOVA of Lisboa, 2829-546 Lisboa, Portugal; gigrejas@utad.pt; 4 Department of Genetics and Biotechnology, University of Trás-os-Montes and Alto Douro, 5000-801 Vila Real, Portugal; 5 Functional Genomics and Proteomics Unit, University of Trás-os-Montes and Alto Douro, 5000-801 Vila Real, Portugal; 6 Institute of Agricultural and Environmental Research and Technology (IITAA), University of the Azores, 9500-321 Angra do Heroísmo, Portugal; sandra.pa.camara@uac.pt; 7 Faculty of Agricultural and Environmental Sciences, University of the Azores, 9500-321 Angra do Heroísmo, Portugal

**Keywords:** lactic acid bacteria, food-producing animals, dairy products, health benefits, one health, antimicrobial resistance, probiotics, starter cultures, adjunct cultures, protective cultures

## Abstract

Animal products, in particular dairy and fermented products, are major natural sources of lactic acid bacteria (LAB). These are known for their antimicrobial properties, as well as for their roles in organoleptic changes, antioxidant activity, nutrient digestibility, the release of peptides and polysaccharides, amino acid decarboxylation, and biogenic amine production and degradation. Due to their antimicrobial properties, LAB are used in humans and in animals, with beneficial effects, as probiotics or in the treatment of a variety of diseases. In livestock production, LAB contribute to animal performance, health, and productivity. In the food industry, LAB are applied as bioprotective and biopreservation agents, contributing to improve food safety and quality. However, some studies have described resistance to relevant antibiotics in LAB, with the concomitant risks associated with the transfer of antibiotic resistance genes to foodborne pathogens and their potential dissemination throughout the food chain and the environment. Here, we summarize the application of LAB in livestock and animal products, as well as the health impact of LAB in animal food products. In general, the beneficial effects of LAB on the human food chain seem to outweigh the potential risks associated with their consumption as part of animal and human diets. However, further studies and continuous monitorization efforts are needed to ensure their safe application in animal products and in the control of pathogenic microorganisms, preventing the possible risks associated with antibiotic resistance and, thus, protecting public health.

## 1. Introduction

Lactic acid bacterial (LAB) are an informal group of prokaryotes that share certain distinctive morphological, metabolic, and physiological characteristics. They are non-respiring, aerotolerant, catalase-negative, fastidious cocci, or rods. Most LAB are non-motile, non-sporulated, may grow in high salt concentrations, and at a low pH [1,2]. Their ancestors were probably soil bacteria, similar to the *Bacillus* genus, which evolved by adapting to nutrient-rich environments, accumulating, in the process, multiple auxotrophies that rendered them fastidious [3,4]. Thus, they have complex nutritional requirements and need several growth factors, such as purines, pyrimidines, vitamins, and amino acids for their growth and metabolic activities. LAB obtain energy by fermenting carbohydrates (e.g., glucose). Based on the pathway for glucose fermentation, LAB are classified as homofermentative, which produce exclusively lactic acid as their final fermentation product, and heterofermentative, which produce acetic acid, CO_2_, and ethanol, in addition to lactic acid [1,2,5]. A recently described group—the fructophilic LAB, present in fruits, flowers, and fermented foods—produce lactate, acetic acid, CO_2_, and ethanol when using fructose as a substrate [6].

The LAB are a genetically and ecologically diverse, but functionally related, group of genera belonging to the *Lactobacillales* order of the *Firmicutes* phylum and encompassing the *Aerococcaceae*, *Carnobacteriaceae*, *Enterococcaceae*, *Lactobacillaceae* (including those formerly part of the *Leuconostocaceae*), and *Streptococcaceae* families. In 2015, 40 bacterial genera were included in this group [7]; a recent revision of the taxonomy of the *Lactobacillaceae*, with the reclassification of the *Lactobacillus* genus giving rise to 23 new genera, substantially increased this number [8]. Although they belong to the *Actinobacteria* phylum, *Bifidobacteria* have been included in the LAB group by some authors, because both have probiotic potential and occupy similar ecological niches [9].

LAB are predominantly found in nutrient-rich habitats. They are part of the normal microbiota of the gastrointestinal tract (GIT) and of the vagina of animals and humans, and they constitute an important element of the non-starter microbial communities found in dairy products (e.g., milk, cheeses, kefir), fish, meat, and vegetables [1,2,6,10,11,12]. For instance, *Oenococcus oeni* plays a significant role in wine fermentation [13], whereas *Lactiplantibacillus plantarum* and *Lactiplantibacillus pentosus* are important for table olive fermentation [14]. On the other hand, LAB have been applied as starter/adjunct/protective cultures in dairy products (e.g., yogurt, cheeses), fermented meats, fermented vegetables, and fermented fish products [15,16,17,18,19,20,21]. They influence the organoleptic and nutritional characteristics of these foods, contributing to their distinctive flavors [11,22].

The unique characteristics that LAB have demonstrated in different biotechnological processes have fueled decades of research by the scientific community. This ongoing research effort has shown the importance of their ability to produce antimicrobial compounds for the control of pathogens, health-promoting applications, the process of food preservation, and agroindustry [5,10,11]. Antimicrobial peptides, such as bacteriocins, produced by LAB have been used against pathogenic microorganisms involved in distinct infections and to control food fermentations [22]. Bacteriocins can be produced directly in the food through the starter, adjunct, and/or bioprotective cultures, or they can be incorporated as preservatives, with the aim to improve food safety and quality [1,10,22]. LAB also hold promise as probiotics, which can aid in the management of pathological conditions, and as therapy adjuvants during the oral intake of antibiotics by modulating the intestinal microbiota of the patients [23,24,25,26].

Several studies have described the positive effects of LAB on human and animal health, as well as in food industry and agriculture [2,6,10]. In foods, LAB contribute to preservation and innovation [2,26]. In humans, the daily consumption of fermented foods and food supplements containing LAB has shown a global increasing trend [26] due to their multiple perceived benefits for human health [2]. The reported beneficial effects of LAB ingestion on human health mainly include the prevention of gut chronic diseases and colon cancer, immunomodulation, promotion of skin health, alleviation of allergic conditions, lactose intolerance, gastroenteritis, diarrhea and peptic ulcer, inhibition of uropathogens, control of plasma cholesterol levels, and production of neurotransmitters (e.g., gamma-aminobutyric acid) [2,10,26,27].

In animals, LAB are also employed to obtain health benefits. The gut microbiota of animals can easily undergo dysregulation (dysbiosis) due to stress, medication, and changes in diet. As probiotics, LAB are used in cattle, other ruminants, swine, poultry, and in beekeeping to improve overall animal health and enhance growth, reproductive performance, and disease resistance [6].

Many studies have reported a diversity of LAB applications in the control of infectious diseases, both in animals and humans, due to their significant antimicrobial activity and probiotic properties. These antimicrobial effects have also been applied to control and inhibit foodborne microorganisms, promoting the safety and preservation of foods and feeds. However, few studies have investigated the impacts of antimicrobial-resistant LAB emergence in animal products on humans and on the environment [28,29]. The main aim of this review is, therefore, to summarize and highlight the application of LAB in livestock and animal products, as well as to discuss the implications of LAB presence in animal products on the health of their human consumers.

## 2. LAB in Livestock Production

In livestock production, LAB are introduced by the diet or as probiotic supplements [6]. These microorganisms, in particular *L. plantarum*, are used as the main crop silage additive that contributes to preserve the nutritional quality of ensilaged forages by inhibiting pathogenic microorganisms and promoting the early onset of lactic acid fermentation. Inoculating forages with homo- and heterofermentative LAB inoculants improved silage fermentation, decreased its final pH, and minimized the production of butyric acid and ammonia-nitrogen, as well as fungal and clostridial growth [30,31]. In addition, encapsulated LAB, such as *Weissella paramesenteroides*, have been applied in biological fish silage for aquaculture production [32].

At any age, perturbations of the native gastrointestinal tract microbiota can have severe consequences for the health, productivity, and development in production animals [33]. To improve animal health by combatting dysbiosis, LAB have been administered to production animals as probiotics, enhancing growth and reproductive performance. In animals, probiotics also show beneficial effects on the treatment or prevention of infectious diseases, can contribute to the reduction in antibiotic use, or be employed as antibiotic alternatives [6,34].

The use of LAB as probiotics in livestock production has been the subject of numerous studies, in which novel strains with different origins and properties have been screened for use in several animal species. Dowarah et al. [35] isolated thirty LAB from piglet feces, most of which showed sensitivity to a variety of antibiotics, such as penicillin, lincomycin, erythromycin, chloramphenicol, and clindamycin, but were resistant to vancomycin and ciprofloxacin. They showed in vitro antibacterial activity against *Klebsiella oxytoca*, *Escherichia coli*, *Salmonella enterica*, and *Staphylococcus intermedius*. In addition, *Pediococcus acidilactici* demonstrated potential probiotic properties in vitro, leading to higher nutrient digestibility, antioxidant activity, and improved hematological profile in weaned piglets [35]. A diet supplemented with *P. acidilactici* not only maintained balance in the gastrointestinal microbiota, but also enhanced the physicochemical properties of swine meat and carcass quality in comparison to *Lactobacillus acidophilus* supplementation [36,37].

Supplementing the diet of lactating Holstein cows with *L. acidophilus* and a non-LAB bacterial species (*Propionibacterium freudenreichii*) induced better nutrient digestibility—in particular, of crude protein and neutral and acid detergent fibers—and increased milk production [38]. Frizzo et al. [39] reported that milk supplemented with probiotic lactic acid bacteria, such as *L. acidophilus*, *L. plantarum*, *Ligilactobacillus salivarius*, *Enterococcus faecium*, *Lacticaseibacillus casei/paracasei*, or *Bifidobacterium* spp., improved feed use efficiency and increased body weight gain in young calves. In addition, *L. acidophilus* reduced diarrhea incidence in calves [38,40]. Another promising potential area of application of direct-fed LAB in ruminant nutrition is in the mitigation of one of the main environmental impacts of milk and meat production—methane emissions [41].

In poultry production, the effects of dietary supplementations with probiotics are well documented [34,42,43]. However, further studies are still necessary to determine the mechanisms of action and assess the optimal dose for multi-strain probiotics. Besides improving the health status of these animals, the use of probiotics can provide an alternative to antibiotic growth promoters, banned by the European Union in 2006, thereby contributing to address a global public health concern—the antibiotic resistance crisis [34,43]. For example, *P. acidilactici* from rectal samples of poultry showed significant antibacterial activities against *E. coli* and *S. enterica* [44]. The applications of LAB that have been selected on the basis of their in vitro immunomodulatory properties to control *Salmonella* infections in broilers proved to be advantageous. Feng et al. [45] reported that LAB with higher in vitro immunomodulatory activities, such as *L. plantarum*, *L. salivarius*, and *P. acidilactici*, were more efficient in achieving a reduction in *Salmonella* counts in the intestinal tract and in minimizing its adhesion to and invasion of broiler livers and spleens. Moreover, broilers fed diets containing *Lactobacillus* showed lower cecal coliform counts, as well as lower cholesterol levels than the control group [42].

A diverse, numerous, LAB symbiotic community is present in the honey crop of bees, defending these important pollinators from invasion by pathogens [46]. The application of LAB as prophylactics in beekeeping could enhance bee health, preventing infectious diseases, possibly contributing to prevent colony collapse disorder and, ultimately, increasing honey yield [11,46].

**Figure 1:**
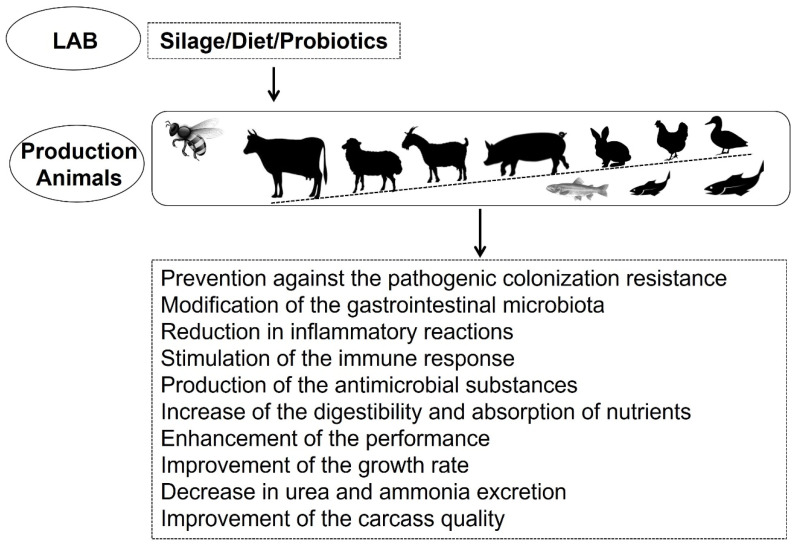
Main advantages of application of lactic acid bacteria (LAB) in animal production.

In general, when applied as probiotics, beneficial LAB have the following positive effects in animals: prevention against colonization by (antibiotic-resistant) pathogens, modulation of the intestinal microbiota, reduction in inflammatory reactions, improvement of carcass traits, modulation of the immune response, performance enhancement, increase in nutrient digestibility and absorption, and decrease in urea and ammonia excretion (Figure 1) [6,34,38,43]. Therefore, dietary probiotics not only have effects on animal health and performance, but they also have potential commercial applications with impacts on the quality of direct products and byproducts resulting from livestock production [36,37,47].

## 3. LAB Applications in Animal Products

**Figure 2:**
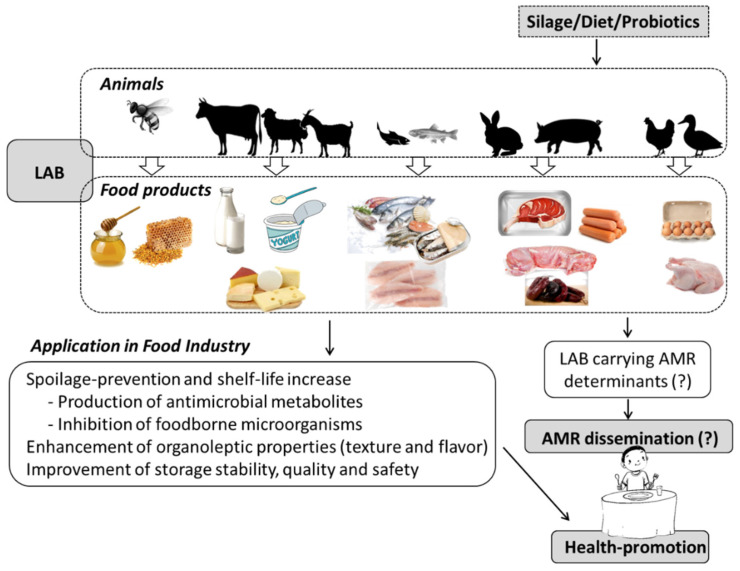
Application of lactic acid bacteria (LAB) in animal products for human consumption and its implications for antimicrobial resistance (AMR) dissemination.

A variety of LAB strains, which are either part of the autochthonous microbiota or introduced into animal products, have potential beneficial applications for the preservation of such products and/or for consumer health (Figure 2). Many foods obtained from fermented products of animal origin, such as meat, fish, and dairy, contain living microorganisms that are phylogenetically similar to probiotic LAB as part of the microbiota that directs their fermentation process and is responsible for their unique character [48]. Fermented foods, such as cultured milk, yogurt, cheese, fermented sausage, and certain types of wine, are obtained through enzymatic reactions resulting from controlled microbial growth, in which the main microbial effectors comprise, primarily, LAB and their metabolites. During the fermentation process, a transformation of the substrate takes place; bioactive or bioavailable metabolic end-products are formed, enhancing the organoleptic (flavor and texture) and nutritional (e.g., vitamin and protein content and bioavailability) properties of the fermentate, and antimicrobial metabolites (such as bacteriocins) can accumulate, reducing the risk of contamination with pathogenic microorganisms and contributing to the preservation of the end-product. In spite of these potentially beneficial results of the fermentative processes, the number of studies that evaluate the health benefits of including fermented feeds in animal diets is still very limited [48,49].

In their fermentative processes, LAB—the main group of bacteria used for food and feed fermentations—release a number of bioactive metabolites, such as biogenic amines, exopolysaccharides, bacteriocins, and other biologically active, proteolytically released peptides [50,51,52]. Hetero- and homopolysaccharides produced by LAB have, respectively, immunomodulatory and prebiotic effects upon their animal hosts, and their industrial application has been studied [53]. LAB are fastidious microorganisms that require the presence of various free amino acids to grow. To ensure the supply of these essential nutrients, LAB possess proteolytic enzymes, bound to their cell walls, which degrade the proteins in the substrate, accumulating peptides and free amino acids. Some of the released peptides have been demonstrated to possess important bioactivities in the host [54]. For instance, bacteriocinogenic strains of LAB can be added to foods as starter cultures, co-cultures, or bioprotective cultures, to improve food quality and safety, as reported in fermented meat and cheese [55,56]. They are also thought to play a role in the potential of LAB as growth promoters for animal production [57]. Besides their antimicrobial activity, peptides released by LAB have been described as possessing immunomodulatory and anti-hypertensive properties [58,59,60,61], as well as activity as defensins [57] and as antioxidants [62].

During food fermentations, LAB are known to decarboxylate amino acids, leading to the accumulation of biogenic amines (BA)—histamine, cadaverine, putrescine, tyramine, and 2-phenylethylamine—under certain environmental circumstances. Enterococci, lactobacilli, streptococci, lactococci, pediococci, and oenococci are regarded as the main BA producers in fermented foods, whereas leuconostocha and weisellae are thought to play a minor role. Decarboxylase activity is, however, a strain-related rather than a species- or genus-related trait [63]. On the other hand, there are reports of biogenic amine degradation by LAB due to their amino oxidase activity [51]. The application of BA degrading LAB could provide a means of controlling the accumulation of these toxins in foods and feeds [64].

Although LAB have demonstrated enormous environmental and health benefits in fermented food products, which could lead to an increase in the demand and consumption of such products, regulation has, in some instances, limited their application [1,65,66]. Some LAB (e.g., the enterococci) are still devoid of qualified presumption of safety (QPS) and generally regarded as safe statuses in the European Union and in the United States of America, respectively [67]. Although genetic determinants of virulence, such as genes encoding host adhesion factors, have been found in these bacteria, it remains to be assessed whether these are virulence traits or merely reflect bacterial adaptations to promote host colonization as part of its beneficial microbiota [68]. The advances in functional genomics will contribute to fill this gap by providing a better understanding of the LAB–food/feed–host interactions [69].

### 3.1. Dairy Products

LAB are ubiquitous in dairy environment and are an important part of the microbiota present in raw milk, fermented milks (such as yogurt, kefir, and viili), and cheeses [2,70,71]. A recent review reported a high LAB diversity isolated from traditional fermented dairy products, such as cheese, yogurt, kajmak, and sour cream, manufactured with bovine, ovine, and caprine raw milk without the addition of a starter culture. From the 28 LAB species isolated, the most prevalent genera were *Enterococcus*, *Lactobacillus* (in its former taxonomic composition), *Streptococcus*, *Lactococcus*, *Leuconostoc*, *Weissella*, and *Pediococcus* [72].

*Lactococcus* spp. are the most frequently isolated LAB in artisanal dairy products [73,74]. In some artisanal raw milk cheeses, lactococci predominate and possibly conduct the maturation processes [72,75,76]. The most commonly found lactococcal species is *Lactococcus lactis* [72,77,78], with the occasional presence of *Lactococcus raffinolactis* and *Lactococcus garviae* [78]. The main role of lactococci in dairy products is to promote acidification by metabolizing lactose, chiefly to L-lactic acid, and to produce flavor compounds (alcohols, ketones, aldehydes) from milk proteins, lipids, and citrate. Acidification is one of the barriers that contribute to the preservation of several dairy products, but certain lactococcal strains also produce bacteriocins that impact on this aspect [79]. Although it is not common, their use as probiotics has been considered [80]. Lactococci bear both GRAS and QPS status. They have been widely used as starter cultures in the dairy industry, particularly *Lac. lactis*, but also, to a lesser extent, *Lac. raffinolactis* [81]. Fermented dairy products constitute an important source of lactococci for the human host, with the estimated annual ingestion of lactococcal cells by human consumers reaching up to 10^8^ cells [82].

Lactobacilli (including all 25 genera in which they have recently been reclassified by Zheng et al. [8]) are frequently isolated from raw milk and fermented dairy products. In most cheeses, lactobacilli are part of the native microbiota, with several species represented. Ten lactobacilli species have been reported in Portuguese artisanal cheeses, manufactured without the addition of a commercial starter: *L. casei* [83,84], *L. paracasei* [83,84,85,86,87], *Latilactobacillus curvatus* [84], *L. plantarum* [84,85,86,87], *L. acidophilus*, *Lactobacillus delbrueckii* [84], *Lentilactobacilus otakiensis* [83,85,86], *Levilactobacillus brevis*, and *Limosilactobacillus fermentum* [84]. Lactobacilli, for example, *L. paracasei*, *Lacticaseibacillus rhamnosus*, *L. plantarum*, and *L. curvatus*, play a role in cheese ripening [73]. In other fermented dairy products, a variety of lactobacilli have also been reported, such as *L. delbruecki* in yogurt [73] and *Lentilactobacillus kefiri*, the most representative of the former *Lactobacillus* species in kefir, a fermented milk drink from the Caucasus Mountains. The kefir grain includes other lactic acid bacteria, such as *L. brevis*, *L. paracasei*, *Lactobacillus helveticus*, *Lactobacillus kefiranofaciens*, *L. plantarum*, *L. kefiri*, *Lac. lactis*, *Streptococcus salivarius* subsp. *thermophilus*, and *Leuconostoc mesenteroides*, besides acetic acid bacteria and yeasts. During fermentation, the production of lactic acid, ethanol, flavor-generating components, and carbon dioxide are responsible for the unique kefir sensorial characteristics. Kefir demonstrated health benefits on the gastrointestinal microbiota and function, as well as in vitro antimicrobial activity against several pathogens, such as *Staphylococcus aureus*, *Enterococcus faecalis*, *S. enterica*, *E. coli*, *Shigella sonnei*, *Bacillus subtilis*, and *Candida albicans* [48]. *L. delbrueckii* and *L. helveticus* are the main lactobacilli used as starter cultures in the dairy industry. Probiotic properties have been attributed to several lactobacillus species. For instance, Pino et al. [88] isolated 166 LAB from Piacentinu Ennese cheese after 90 days of ripening, indicating *L. paracasei* and *L. rhamnosus* as the main LAB species with promising probiotic properties. Dairy products have been frequently used as carriers for probiotic lactobacillus strains, such as *L. acidophilus* and *L. rahmnosus* [89]. Many, but not all, of the species included in the lactobacillus group of genera possess QPS status [90].

Leuconostocha are part of the core microbiota in several artisanal cheeses, although their populations are, in general, numerically lower than those of lactococci [83]. The main species described in starter free cheeses from Portugal were *Ln. mesenteroides*, *Leuconostoc lactis* [84], *Leuconostoc pseudomesenteroides* [83], and *Leuconostoc citreum* [85]. All these leuconostoc species possess QPS status. *Leuconostoc* spp. participate in the maturation of non-hard cheese varieties, including Camembert, where they may contribute to flavor formation, to their rheological properties by producing exopolysaccharides, and/or to open texture due to CO_2_ production [38,91,92]. *Ln. lactis* and *Ln. mesenteroides* are used in the production of butter, sour cream, and other non-maturated dairy products [91]. Probiotic potential has been described in leuconostoc strains isolated from dairy products [85]. Dairy products may serve as a considerable source of leuconostocha for the human host. Firmesse et al. [92] reported high levels of *Lac. lactis* and *Ln. mesenteroides* in fecal samples during the consumption of Camembert cheese, which, in the case of *Ln. mesenteroides*, persisted 15 days after the end of consumption.

Enterococci are members of the non-starter microbiota in many artisanal cheeses, where their role in flavor development has been documented [67,93]. A total of 19 species have been described in artisanal cheeses so far—*Enterococcus avium*, *Enterococcus casseliflavus*, *Enterococcus devriesei*, *Enterococcus durans*, *E. faecalis*, *E. faecium*, *Enterococcus gallinarum*, *Enterococcus gilvus*, *Enterococcus hirae*, *Enterococcus italicus*, *Enterococcus lactis*, *Enterococcus malodoratus*, *Enterococcus mundtii*, *Enterococcus pallens*, *Enterococcus pseudoavium*, *Enterococcus ratti*, *Enterococcus saccharominimus*, *Enterococcus sulfureus*, and *Enterococcus villorum* [67]. Their potential as starter cultures, however, is much less evident. Not only do they often lack the desirable acidification and proteolytic capacity, but also, none of the species in this genus have yet been granted GRAS/QPS status. The opportunistic potential of enterococci has weighed in this decision. However, food enterococci seem to differ considerably in their virulence from clinical isolates, and further studies might allow the use of certain enterococci in food settings. Dairy enterococci could provide a promising reservoir of bacteriocin-producing strains with the potential to modulate the microbiota of cheeses, that could be used as protective cultures, thereby promoting their safety [67]. The potential of several enterococci as probiotics has been reported, both for human and animal applications, but the aforementioned safety concerns have limited their commercial application [67,94].

Certain streptococci, such as *Strep. salivarius* ssp. *thermophilus* [1] and *Streptococcus gallolyticus* subsp. *macedonicus* [95], are part of the LAB, possess QPS status, and are commonly present in dairy products. *Strep. salivarius* subsp. *thermophilus* is typically used as a starter culture to ferment milk, usually of ruminant origin, giving rise to a diversity of products with unique flavors, textures, and nutritional properties [1]. In yogurts, it is symbiotically associated with *L. delbrueckii*, where this bacterial consortium acidifies the milk and produces acetaldehyde—a key compound for the flavor of this product. In cheeses, it also contributes to acidification of the curd and to flavor formation during ripening. Furthermore, there are reports of exopolysaccharide (EPS) and bacteriocin production by this species, as well as probiotic characteristics [96].

Dairy products can act as reservoirs of lactic acid bacteria for their human consumers. The lactic acid bacteria in these products may impact human health, with several species included in this group presenting potential as protective and/or probiotic cultures. Bacteriocin production is of particular interest in this respect. Pathogenic microorganisms found in raw milk, such as *Staph. aureus*, *E. coli*, *Listeria monocytogenes*, and *Salmonella* sp., can be transferred from livestock animals to humans through the consumption of milk and dairy products, causing severe diseases. Although the bacterial contaminants in milk are largely eliminated by the pasteurization process, the application of LAB producing bacteriocin-like compounds in fermented and nonfermented dairy products could improve their quality and safety, decreasing the associated food-borne infection risks [97]. The bacteriocin-type compounds (e.g., lactocidin, acidolin, and acidophilin) produced by *L. acidophilus* have a broad spectrum of inhibitory activity against species of Gram-negative and Gram-positive bacteria (*Salmonella*, *Shigella*, *Clostridium*, *Staphylococcus*, *Listeria*), yeasts (*Candida*), and protozoa (*Trypanosoma*) in dairy products [1]. Moreover, lacticin produced by *Lac. lactis* inactivated *L. monocytogenes* in cottage cheese and yogurt. Nisin, the best known of the bacteriocins produced by *Lac. lactis*, demonstrated bactericidal activity against *L. monocytogenes* and *Clostridium* in milk, preventing the spoilage process known as late blowing in cheese [97,98]. A recent study reported that the major bioprotective mechanism responsible for the inhibition of deteriorative microbiota in fermented dairy products by members of the former *Lactobacillus* genus is through competitive exclusion by expression of the *mntH1* gene. This novel and natural mechanism was demonstrated to inhibit the growth of spoilage microorganisms, such as molds and yeasts, in dairy products (e.g., yogurts) [99]. It can be concluded that autochthonous dairy LAB exhibit unique technological and health-promoting properties, including probiotic activities, production of flavor compounds, bacteriocins, and other bioactive peptides, demonstrating their potential for the production of novel, health-promoting foods [72].

### 3.2. Meat and Fermented Meat Products

Meat products harbor a diverse LAB microbiota, in which lactobacilli (*Latilactobacillus sakei*, *L*. *curvatus*, and *L*. *plantarum*) usually predominate [100] and play an important role in maturation. In certain cases, considerable populations of leuconostocha (*Ln*. *mesenteroides*, *Leuconostoc carnosum*) and enterococci (*E*. *casseliflavus*) are also present [101,102]. Meat fermentation is a complex process, from the point of view of its microbial ecology, in which both LAB and coagulase-negative staphylococci intervene, participating in the development of the typical sensorial properties of the product and in its biopreservation [103].

LAB can either be used as meat starter cultures and/or as probiotics, interacting with native microorganisms in the product, or they can be part of the non-starter microbiota in fermented products. In both cases, their presence can have potential advantages for the end-products [104,105]. Fermentation allows the preservation of meat products and the production of a variety of fermented meats with different organoleptic characteristics as a result of the microbial and endogenous enzymatic reactions taking place within their primary component—the animal muscle. The use of starter cultures, including probiotic microorganisms with health-promoting potential, such as the ability to reduce cholesterol content, contributes to the promotion of product stability and safety, as well as consumer acceptance [105,106,107].

Several criteria should be considered to select LAB for the production of fermented meats. The ability to acidify and grow at low pH values are desirable factors for potential starter cultures for the meat industry and for spoilage prevention, because they lead to increased safety and prolonged shelf life of the final products by inhibiting the growth of pathogenic and deterioration microorganisms, facilitating maturation, ensuring microbial stability during storage, stabilizing the product color, and improving its texture. Another desirable trait is proteolytic activity, which plays an important role in flavor development during the fermentation process, as is the case in raw sausage fermentation. During meat fermentation, LAB can positively influence protein degradation. The resulting peptides can be further converted into volatile compounds, which is important for the sausage flavor [105,106,107]. Proteolytic activity, acidification, and the ability to produce low final pH values were observed in LAB, particularly in lactobacilli, isolated from Bulgarian traditional fermented lulanka salami, manufactured from veal, pork, and spices encased into dried bovine intestines [105]. The analysis of the most popular dry fermented sausages in Spain, chorizo and salchichón (which mainly included minced pork and deer meat), demonstrated that the final pH value attained, and the amount of lactic acid produced, influenced microbial counts in these meat products [108]. The antimicrobial activity of LAB is another desirable trait, since inhibition of the proliferation of spoilage bacteria and foodborne pathogens is important to ensure product quality, shelf life, and safety [107]. Todorov et al. [105] reported the presence of antimicrobial activity in *L. plantarum* and *L. brevis* from traditional salami against *L*. *monocytogenes*, an additional advantage for the product biopreservation. Moreover, antimicrobial activity against other tested LAB, such as *Lactococcus* spp., *Pediococcus* spp., and *Enterococcus* spp., was not observed. In addition, few strains in this study showed the presence of genes for virulence factors, biogenic amine production, and resistance to vancomycin. On the other hand, a high prevalence of bacteriocin genes was detected. Other beneficial properties to take in account when screening LAB to add into fermented meat products include the ability to degrade biogenic amines [109], cholesterol [110], and carcinogens in meats [111], particularly in smoked meat products [112], as well as the capacity to control lipid oxidation [112].

Several of the LAB associated with fermented meats are bacteriocinogenic and possess, therefore, the ability to modulate the microbiota of the product, combatting the proliferation of pathogenic (*L*. *monocytogenes*, *E*. *coli* O157:H7, *Salmonella* spp., and *Clostridium botulinum*) and deterioration microorganisms [113]. Meat products often rely on hurdle technology for their safety and preservation; bacteriocins can, in this context, provide an additional hurdle. Bacteriocinogenic *L*. *curvatus* has been successfully employed to extend the shelf life and improve microbial safety in meats, effectively controlling *L*. *monocytogenes* and *Brochothix thermosphacta* in fresh beef, without negatively impacting its sensory properties [114]. Leroy et al. [115] demonstrated that *L*. *sakei* isolated from sausage produce the sakacin K bacteriocin, that is active against some listerial strains, leading to inactivation of *Listeria innocua* during the sausage fermentation process. In another study, 813 bacteriocin-producing LAB strains that demonstrated the ability to inhibit the growth of *L*. *innocua* and *Staph. aureus* were isolated from 174 meats and meat products [109]. Of these, five bacteriocin-producing strains, including *Lac*. *lactis* and *L*. *plantarum*, were analyzed in greater detail. All of them displayed traits consistent with probiotic potential, such as tolerance to very low pH, survival/growth at gastrointestinal tract and at food storage temperatures, as well as biofilm production [48]. Although the probiotic potential of autochthonous meat LAB has been demonstrated, fermented meat products are not a common vehicle for probiotic bacteria. The conditions within their matrices (pH values, water activity, inhibitory concentrations of H_2_O_2_, organic acids, sugars, and additives) have a negative effect on probiotic viability. However, the selection of the adequate strains and/or techniques such as microencapsulation might help probiotic LAB survive under these conditions, enabling their addition to fermented meats [116,117].

In certain cases, LAB have been associated with deteriorative processes in meats. Certain carnobacteria, lactobacilli, leuconostocha, weissellae, and lactococci can lead to acidification, produce off-odors, blowing of packages, slime, and green discoloration in fresh meats [118]. Regarding their antibiotic resistance/sensitivity profile, resistance to erythromycin was common; resistance to vancomycin, chloramphenicol, tetracycline, and chloramphenicol was present in a few strains [119].

### 3.3. Fish and Fishery Products

Fish and seafood are an important part of the human diet. These foods harbor a numerically low, but diverse, community of authochthonous LAB, in which lactobacilli, lactococci, leuconostocha, enterococci, streptococci, carnobacteria, weissellae, and pediococci have been identified [120]. For instance, *Companilactobacillus farciminis*, *L. sakei*, and *Companilactobacillus alimentarius* were identified in smoked trout, cooked cold-water shrimp, and cold-smoked salmon [121].

Fish and seafood can spoil very rapidly; the required hygienic quality and safety standards are, therefore, difficult to maintain. Due to the production of antimicrobial metabolites, such as organic acids and bacteriocins, LAB can be regarded as natural biopreservative agents, although scarce commercial applications of these bacteria have been developed from/for fish and fishery products [121]. LAB of marine origin and their metabolites possess, however, a major potential as fish biopreservatives, due to the abundance of antimicrobial mechanisms they are able to deploy: acidification, bacteriocins, lysozymes, proteases, siderophores, and/or hydrogen peroxide production [121]. Furthermore, they are abundant in the microbiota of many fish species, and most LAB genera are recognized as safe [21,122]. The application of LAB biopreservative potential in fish and fishery products has been demonstrated in several works. For instance, the addition of *L. curvatus* and *E. faecalis*, through the production of their bacteriocins, sakacins, and enterocin, respectively, was shown to improve the organoleptic properties, inhibit *L. monocytogenes*, and increase the market value of young hake and megrim. *L. sakei* also showed activity against *L. monocytogenes* [51]. Additionally, Sarika et al. [123] demonstrated that bacteriocin PSY2 from *Lac. lactis*, isolated from marine perch, provided an extended shelf-life and better protection against spoilage bacteria in reef cod fillets. Autochthonous LAB belonging to the *Aerococcus*, *Carnobacterium*, *Leuconostoc*, and *Vagococcus* genera, applied in conjunction with chitosan, modified atmosphere packaging, and superchilling, proved to be an efficient additional hurdle against several fish deteriorating bacteria (*Shewanella baltica*, *Photobacterium phosphoreum*, *B. thermosphacta*, *L. sakei*, *Hafnia alvei*, and *Serratia proteamaculans*) [124]. The application of LAB to fishery products as biopreservative cultures could, therefore, provide additional support for preservation and safety assurance in these highly perishable commodities. As in other food commodities, bacteriocin production is an important trait in the screening for LAB destined to be used as biopreservatives for fishery products [21].

Fermented fish products are not an important component of Western diets. In Asia, however, traditional fermented fish products, such as plaa-som, hoi-dorng, som-fak, pa-ra, pla-chom, kung-chom (Thailand), bekasam, chao (Indonesia), chouguiyu (China), burong baugus, burong isda (Philippines), budu, pekasam (Malaysia), ngari, hentak, and tungtap (India), may constitute important sources of dietary protein [125]. Fermentation also provides a means of upgrading fish waste and by-catch, an important issue in an industry that discards up to 70% of its captures [51,126,127]. The resulting fermentate can be utilized as feed for certain animal species [51,126], as a protein hydrolysate for microbiological culture media [128], or as fertilizer for organic farming [129]. Besides the technological properties required for the fermentation of other food matrices, LAB must also be able to limit the accumulation of biogenic amines in fermented fish products. These microbial metabolites are of particular concern in fish species (such as those belonging to the *Scombridae* family) that are rich in their precursor amino acids [51].

### 3.4. Other Animal Products

The application of dietary LAB in apicultural and poultry production has demonstrated promising effects on animals, their products, and their human consumers. Mikulski et al. [47] reported that a diet supplemented with *P. acidilactici* in the early laying phase improved the market value of carcasses and eggshell quality. A significant decrease in the number of broken shells, absent shells, and downgraded eggs, as well as an increase in egg weight, relative weight, and thickness of the eggshells were observed. In addition, probiotic supplementation improved feed efficiency ratio and hen performance. However, the main objective in studies on probiotic application in egg production has been to reduce the cholesterol content of this product. Several studies have demonstrated evidence that LAB supplementation reduces cholesterol content in the egg yolk [130,131,132], with Mikulski et al. [47] and Haddadin et al. [130] reporting a reduction of more than 10 and 18%, respectively, with the addition of *P. acidilactici* and *L. acidophilus*. In these studies, egg yolk cholesterol content depended on the administered dose and on the bacteria used as probiotics.

Honeybees are distributed worldwide, and besides producing honey, these insects are considered one of the main crop pollinators. The products and byproducts resulting from apicultural production, such as honey and pollen, are known for their medicinal and nutritional properties, and are classified as safe, health-promoting foods [4]. Within the LAB, the former *Lactobacillus* genus was the most frequently identified in the beehive, and *Apilactobacillus kunkeei* was predominantly isolated from honey, bee pollen, bee bread, and royal jelly. Immunomodulatory activity through the secretion of IgA has been reported in this species [133]. In another study, a total of 43 LAB species was identified in beehives, and of these, 20 species showed antimicrobial activity against animal and human pathogens [6]. Lactobacilli, *Enterococcus*, and *Weissella* demonstrated in vitro inhibitory effects against *Paenibacillus larvae*, an enthomopathogen that affects bee larvae [134]. Moreover, honey has demonstrated antimicrobial properties against a broad range of pathogenic bacteria, including *Staph. aureus*, *Pseudomonas aeruginosa*, *E. coli*, *Bacillus cereus*, and *Salmonella* spp. [135]. Not only may the use of probiotics containing LAB species in beekeeping contribute to prevent bee diseases and increase honey production, but also the promising antimicrobial characteristics against pathogenic microorganisms of the native LAB found in honey and other bee products can make them suitable candidates for the development of food-grade biopreservatives [6,135]. However, antibiotic resistances were also evident in some of these LAB, a factor that must be considered prior to their application [6].

## 4. Presence of Antimicrobial-Resistant LAB in Animal Food Products

Measures have been implemented worldwide to limit the inadequate use and overuse of antibiotics in animals, particularly in livestock production. The increase in antimicrobial resistance to antibiotics in animal production chains and the presence of antibiotic residues in animal products can pose serious health risks for humans as final consumers of these products [42]. Probiotics have increasingly been applied as an alternative to antibiotics, both as animal growth-promoters and in the combat against pathogenic microorganisms. LAB with health-promoting abilities are usually classified and used as probiotics. Although the benefits of using LAB in the production of a variety of dairy and meat fermented foods are well documented, due to their contribution to the sensorial properties of these foods and to the production of antimicrobial compounds, the presence of antimicrobial resistance in LAB strains used in the food industry is not [136].

Most LAB used in animal feed or animal-derived foods occur naturally in the animals (e.g., in their gastrointestinal tract) or in the resulting products. Although LAB with probiotic potential are considered non-pathogenic, the risks associated with the possible genetic transfer of antimicrobial resistance or toxin production to pathogenic microorganisms cannot be disregarded [38,73]. Horizontal transfer of antibiotic resistance genes may occur through mobile elements (integrons, transposons, and plasmids). In some strains of the former *Lactobacillus* genus, the presence of antibiotic resistance genes has been reported [28]. Some LAB, beyond acquired antibiotic resistances, also show intrinsic antibiotic resistance. For example, the best characterized intrinsic resistance in LAB is that of some lactobacilli to vancomycin. The presence of intrinsic antibiotic resistance genes is undesirable but may not constitute a safety issue, since LAB are very rarely involved in infections, and these genes are not easily mobilized and transferred to other pathogens [105].

Several studies on autochthonous food bacteria have highlighted the potential role of LAB as reservoirs of antibiotic resistance genes, from where resistance determinants could be transferred to the human microbiome via food chain [28,137,138,139]. For instance, in the production of dry-fermented sausages, antibiotic-resistant LAB may be introduced through contamination of the raw materials and added ingredients, fecal contamination, improper handling, environmental contamination, or cross-contamination, together with the lack of processing steps that allow the elimination of these microorganisms. The close contact among bacteria in these foods could facilitate the horizontal transfer of the antibiotic resistance genes they carry to pathogenic bacteria or to other LAB during processing [104,140]. The horizontal transfer of resistance genes between LAB ingested with foods and from LAB to other bacteria may also take place in the consumers’ gut [141].

Genetic determinants of tetracycline, vancomycin, and erythromycin resistance have been described in *Enterococcus* spp., *Lac. lactis*, and several lactobacilli from fermented meat and milk products [28,138]. The potential role of enterococci as reservoirs of antibiotic resistance genes in dairy products [140] and their particular propensity to trade these genetic determinants with other bacteria, including pathogens, is well known [142]. A recent study detected widespread resistance to several antibiotics in LAB from fermented dairy products (yogurt and a fermented dairy drink), of which several were lactobacilli (*L. delbrueckii* subsp. *bulgaricus*, *L. plantarum*, *L. paracasei*, and *L. acidophilus*) and *Strep. salivarius* subsp. *thermophilus*. In this study, most of the strains belonging to the former *Lactobacillus* genus were resistant to streptomycin (84%) and gentamycin (84%); less frequently, these strains showed resistance to erythromycin, sulfamethoxazole, and tetracycline. Most *Strep. salivarius* subsp. *thermophilus* also harbored resistance genes to the aminoglycosides streptomycin (92%) and gentamycin (87%), as well as to ciprofloxacin (79%), chloramphenicol (72%), and erythromycin (8%) [143]. In another study, *L. plantarum* isolated from raw-milk cheese showed erythromycin resistance [144]. The research on antibiotic resistance in probiotic microorganisms obtained from yogurts, yogurt-type fermented milk, and pharmaceutical products detected resistance to aztreonam, cycloserin, kanamycin, nalidixic acid, polymyxin B, and spectinomycin in all LAB strains belonging to the *Streptococcus* and former *Lactobacillus* genera, as well as in bifidobacteria [145]. Additionally, it has been demonstrated that *L. delbrueckii* subsp. *bulgaricus* and *L. plantarum* can successfully transfer the *tetM* and *tetS* tetracycline resistance genes to *L. monocytogenes* [143].

In meat products, all strains of *Lac. lactis* and *L. plantarum* demonstrated in vitro resistance to erythromycin, and some were also resistant to vancomycin, tetracycline, and chloramphenicol, as previously described [119]. Antibiotic resistance to vancomycin was also detected in LAB strains isolated from traditional salami [105]. Gevers et al. [140] analyzed LAB resistance to tetracycline along the process of fermented dry sausage, demonstrating the presence of resistant lactococci, lactobacilli, streptococci, and enterococci in the raw meat, but only resistant lactobacilli were persisted after fermentation. Both the *tet*M and *tet*S tetracycline resistance genes were found in the raw meat isolates, but *tet*(M), found exclusively in the lactobacilli, was the only gene detected after sausage fermentation. This study confirms that raw meat already contains a subpopulation of resistant bacteria and that the resulting fermented meat can act as a vehicle for tetracycline-resistant LAB. However, Jha et al. [43] reported that probiotic use can reduce subtherapeutic antibiotic use in the poultry production and mitigate public health concerns with antibiotic resistance transfer via animal products. Additionally, there is not enough evidence that antibiotic resistance genes are transferred in poultry production by probiotic supplementation [43]. Therefore, at present, the benefits of antibiotic supplementation in food producing animals seem to outweigh the potential risks associated with the dissemination of antibiotic resistance genes. However, evaluating the resistome of LAB intended for food purposes is necessary to mitigate the dissemination of such genetic determinants to human communities, mediated by foods of animal origin.

## 5. Implications of Foodborne Antibiotic-Resistant LAB in Human Health: One Health Perspective

With the surge in human population and the rising threat of antibiotic resistances, the necessity to promote research on alternatives to antibiotics has also increased. LAB use, especially as probiotics, are becoming popular, and they have, indeed, been proven useful, both for humans and animals [2,38]. LAB application in feed improves animal performance and health, preventing infections by enteric pathogens. Moreover, LAB reduce bacterial contamination through the inhibition of foodborne pathogens in animal products, enhancing the safety and quality of the end-products in the food industry. However, this does not mean that other preventive measures—such as good hygio-sanitary practices and hygiene monitorization of the raw materials—should be neglected [98]. In humans, LAB promote health and show several beneficial effects, e.g., a reduction in lactose intolerance, maintenance of normal insulin level, antidiarrheal, antineoplastic, and anti-inflammatory activity, among others [2]. However, there is limited information on the risks of probiotic bacteria used in animals and, consequently, of their products, for the human consumer and the environment. The major associated risk is the possible transference of antibiotic resistance genes from probiotic LAB strains to other bacteria [38,73]. In addition, the risks associated with probiotics also comprise the possibilities of infection and immune hyper-stimulation in consumers of animal products containing probiotic bacteria. Although infrequently reported, concerns with infection and sensibilization of skin, eye, and mucous membranes in probiotic handlers, as well as environmental contamination with potentially pathogenic microorganisms or noxious compounds, have been described [38,146,147].

With the exception of the enterococci, LAB infections are extremely rare. However, some cases of bacteremia, endocarditis, lactobacillemia, and antibiotic-resistant infections have been reported in severely immunocompromised individuals [73]. It must be taken in account that, in the light of current knowledge, LAB beneficial effects outweigh their rare harmful effects, such as the evidence that LAB may act in the food chain as reservoirs of antimicrobial resistance genes that are horizontally transmissible to other bacteria. Indeed, according to Masood et al. [2], a routine human diet should be rich in LAB. In order to safeguard public as well as environmental health, FAO considers that there is a need for better regulation and guidelines on the use of LAB probiotics [38]. It is important to detect and assess antibiotic resistance expression, as well as to evaluate the real risk of the presence of antibiotic resistance genes in LAB from animal products and in the human gastrointestinal tract, so that specific management strategies can be adopted throughout the food chain to mitigate the potential dissemination of antibiotic resistance through animal food products [104].

On the other hand, more information is required on potential non-food uses of LAB with inhibitory potential or antimicrobial activity against human and animal pathogens, that could aid in limiting medical antibiotic use. For instance, a high number of pathogens implicated in urinary tract infections have demonstrated antibiotic resistance, making the treatment of these infections more difficult. LAB demonstrated, in vitro, the ability to inhibit uropathogens [10] and their use for this purpose could, therefore, be envisaged. Although they have mostly been used as probiotics or starter or adjunct cultures, and their bacteriocins have been exploited as food preservatives, novel and emerging applications of LAB to inhibit human pathogens as bioprotective agents and therapeutical adjuvants, or as oral vaccines, could be anticipated for the prevention and treatment of human diseases [10], thereby contributing to minimize the use of antibiotics.

## 6. Conclusions

Animal products, in particular dairy products, are natural and major sources of LAB with several beneficial effects for humans and animals. LAB can be advantageously applied in animal production to improve animal performance, health, and productivity; in the food industry, they have demonstrated potential as biopreservatives due to their antimicrobial properties. Their consumption in animal products has been demonstrated to have beneficial effects on human health, although a residual risk of antibiotic resistance transfer through these foods exists. This review demonstrated that, under the light of the present knowledge, the beneficial effects of LAB on the human food chain outweigh their potential harmful impacts. However, further studies and a continuous monitorization effort are necessary for the safe application of LAB in animal food products and in the treatment of pathogenic microorganisms, providing a thorough assessment of the possible risks associated with the dissemination of antimicrobial resistance genes and, thereby, protecting public health.

## Data Availability

Not applicable.
